# Comparison of response to 2-years’ growth hormone treatment in children with isolated growth hormone deficiency, born small for gestational age, idiopathic short stature, or multiple pituitary hormone deficiency: combined results from two large observational studies

**DOI:** 10.1186/1687-9856-2012-22

**Published:** 2012-07-12

**Authors:** Peter A Lee, Lars Sävendahl, Isabelle Oliver, Maithé Tauber, Oliver Blankenstein, Judith Ross, Marta Snajderova, Viatcheslav Rakov, Birgitte Tønnes Pedersen, Henrik Thybo Christesen

**Affiliations:** 1 Penn State College of Medicine, The Milton S. Hershey Medical Center, PO Box 850, Hershey, PA, 17033-0850, USA; 2 Division of Pediatric Endocrinology, Department of Women’s and Children´s Health, Karolinska Institutet, Stockholm, Sweden; 3 Division of Pediatric Endocrinology, Hôpital des Enfants, Toulouse, France; 4 Department of Pediatric Endocrinology, Charité-Universitätsmedizin Berlin, Berlin, Germany; 5 Department of Pediatrics, Thomas Jefferson University, Philadelphia, PA, USA; 6 Department of Pediatrics, 2nd Medical School - Charles University and University Hospital Motol, Prague, Czech Republic; 7 Novo Nordisk Health Care AG, Zurich, Switzerland; 8 Novo Nordisk A/S, Søborg, Denmark; 9 H.C. Andersen Children’s Hospital, Odense University Hospital, Odense, Denmark

**Keywords:** Isolated growth hormone deficiency, Small for gestational age, Multiple pituitary hormone deficiency, Idiopathic short stature, Puberty, Norditropin®

## Abstract

**Background:**

Few studies have compared the response to growth hormone (GH) treatment between indications such as isolated growth hormone deficiency (IGHD), born small for gestational age (SGA), idiopathic short stature (ISS), and multiple pituitary hormone deficiency (MPHD). The aim of this analysis of data, collected from two large ongoing observational outcome studies, was to evaluate growth and insulin-like growth factor-I (IGF-I) response data for children of short stature with IGHD, MPHD, SGA, or ISS following two years of treatment with the recombinant GH product Norditropin® (Novo Nordisk A/S, Bagsværd, Denmark).

**Methods:**

Analysis of auxologic data from two ongoing prospective observational studies, NordiNet® International Outcomes Study (NordiNet® IOS) and NovoNet®/American Norditropin® Studies: Web-enabled Research (ANSWER) Program®.

**Results:**

4,582 children aged <18 years were included: IGHD, n = 3,298; SGA, n = 678; ISS, n = 334; and MPHD, n = 272. After two years’ GH treatment, change in height standard deviation score (SDS) was +1.03 in SGA and +0.84 in ISS vs. +0.97 in IGHD (*p =* 0.047; *p <* 0.001 vs. IGHD, respectively). Height gain was comparable between IGHD and MPHD. In pre-pubertal children vs. total population, height SDS change after two years was: IGHD, +1.24 vs. +0.97; SGA, +1.17 vs. +1.03; ISS, +1.04 vs. +0.84; and MPHD, +1.16 vs. +0.99 (all *p* < 0.001).

**Conclusions:**

After two years’ GH treatment, change in height SDS was greater in SGA and less in ISS, compared with IGHD; the discrepancy in responses may be due to the disease nature or confounders (i.e. age). Height SDS increase was greatest in pre-pubertal children, supporting early treatment initiation to optimize growth outcomes.

## Background

Growth hormone (GH) treatment is approved for treatment of short stature in a number of childhood diagnoses, such as isolated growth hormone deficiency (IGHD) and multiple pituitary hormone deficiency (MPHD). There are other childhood indications, which are not associated with a deficiency of endogenous growth hormone that can be improved by GH treatment, for instance, born small for gestational age (SGA) and idiopathic short stature (ISS) [1,2]. Growth hormone treatment has been shown to increase final adult height in each of these patient populations [3-12]. Moreover, in patients with IGHD, total gain in height SDS was reported to correlate significantly with pre-pubertal gain in height SDS, with younger age at treatment start being a significant predictor of greater treatment response [13,14].

Few studies have directly compared the growth response to GH treatment according to diagnosis and pubertal stage. Some studies suggest that pre-pubertal children with ISS experience less gain in height with GH treatment than short children with growth hormone deficiency [15,16]. The growth response has, however, not been compared in large, observational real-life studies across indications and pubertal stage.

Treatment outcomes that can be achieved in daily clinical practice may differ widely from the more controlled setting of randomized trials; therefore, post-marketing surveillance studies are required to generate data from large cohorts about the efficacy (and/or safety) of an intervention in the real-world clinical setting, which may then be used to inform changes in indications. While there are obvious advantages to the data provided by observational studies, there are also a number of drawbacks that arise from the necessity of having a heterogeneous, all-comer study population; these include, but are not limited to, non-standardization of laboratory tests, treatment dose and number of injections, missing parameters (e.g. mid-parental height, target height, birth weight), and variation in local practices when studies are multinational.

The aim of this analysis of data, collected from two large ongoing observational outcome studies, was to evaluate growth and insulin-like growth factor-I (IGF-I) response data for children of short stature with IGHD, MPHD, SGA, or ISS following two years of treatment with the recombinant GH product Norditropin® (Novo Nordisk A/S, Bagsværd, Denmark). A secondary focus was to assess the impact of GH therapy in pre-pubertal children compared with the total patient population within each of the indications investigated.

## Methods

Data for this analysis were obtained from the NordiNet® International Outcome Study (IOS; NCT00960128) launched in 2006 and ongoing in 19 countries (Czech Republic, Denmark, Finland, France, Germany, Hungary, Ireland, Israel, Italy, Lithuania, Montenegro, Netherlands, Norway, Russia, Serbia, Slovenia, Sweden, Switzerland, UK), and from the US observational study NovoNet®/American Norditropin® Studies: Web-enabled Research (ANSWER) Program® (NCT01009905), which began in 2002 and is also ongoing. The IOS and the ANSWER Program® use a similar electronic platform, NordiNet®/NovoNet®, to collect and manage data about the effectiveness and safety of Norditropin® in normal clinical practice. Physicians enter data on patient history, physical examinations, and treatment regimens using the web-based NordiNet®/NovoNet® tool.

The use of Norditropin® for patients included in these large observational studies is at the discretion of the participating physicians as part of their routine clinical practice. Both observational studies are operated in accordance with the Declaration of Helsinki and with the approval of local institutional review boards.

Data are anonymized in the two observational studies. For all patients, physicians measure a number of variables at the initial visit according to their standard medical practice, including baseline height, weight, bone age, Tanner stage or testicular volume, maximum stimulated serum GH concentration, and serum IGF-I concentrations. At follow-up visits, data gathered include GH dose and injection frequency, height, weight, Tanner stage or testicular volume, and IGF-I levels, among other variables.

For this analysis, only patients aged <18 years with data collected at baseline AND at both one and two year follow-up visits (±3 months) were included. Data were divided by indication based on investigators’ clinical diagnosis (IGHD, MPHD, SGA, and ISS), gender, and pubertal status. Patients with IGHD were used as the reference group because this population represents the optimal indication for replacement GH treatment, where it is unlikely that other hormone deficiencies act as confounders for outcomes; in addition, there exists a large quantity of efficacy and safety data for GH treatment in IGHD. For this study, the pre-pubertal population group was defined using descriptions of clinical puberty symptoms: girls had Tanner stage 1 breast development, while boys had a testicular volume <4 mL. Children were identified as pre-pubertal when they remained at this stage of development for the entire two-year follow-up period. To be identified as pre-pubertal when information on pubertal status was lacking, girls had to be no more than 6 years of age, while boys had to be no more than 7 years old at the start of the observational period; this ensured that patients with missing information were unlikely to start puberty during the observational period, because mean age −2 SD at start of puberty in the observed patient population was ~8 years for girls and ~9 years for boys.

For all patients included in this analysis, height SDS at baseline and change in height SDS at two years were calculated. For patients in the NovoNet®/ANSWER Program® study, height SDS was determined according to standard formulas provided by the Center for Disease Control and Prevention [17], and for patients in the NordiNet® IOS study by the corresponding country references. Bone age was determined manually or with the automatic software application BoneXpert®, which is included in the NordiNet®, but not in the NovoNet® system. When laboratory IGF-I measurements were available, IGF-I SDS values were calculated according to established age- and sex-specific reference values and models [18] without adjustments for local differences in laboratory procedures or possible variations in IGF-I levels.

### Statistical analysis

Descriptive statistics and a simple analysis of variance (ANOVA) model were used to analyze combined patient data from the NordiNet® IOS and the NovoNet®/ANSWER Program®. Variables examined included change in height SDS and change in IGF-I SDS. Analyses were made both on the total population and on the sub-population of pre-pubertal patients within each indication. No adjustment for multiplicity of testing was done. Differences were regarded as statistically significant if the *p*-value was <0.05. All analyses were performed with SAS software, version 9.1 (SAS Institute Inc., Cary, NC, USA).

## Results

### Baseline characteristics

NordiNet® IOS and NovoNet®/ANSWER Program® have yielded data on >11,000 and >11,500 Norditropin®-treated patients, respectively, most of whom were children with a variety of diagnoses, treated for short stature. For this analysis, 4,582 pediatric patients (i.e. the total population in this analysis) with the following indications and with two years of follow-up data were identified in the databases: IGHD, n = 3,298; SGA, n = 678; ISS, n = 334; and MPHD, n = 272. Mean age at treatment start was 10.2, 7.9, 10.9, and 7.9 years for IGHD, SGA, ISS, and MPHD, respectively (Table 1). Children born SGA had the lowest baseline height SDS of −3.1. Mean bone age was delayed relative to chronological age across all indications, ranging from −1.6 to −1.8 years. With the exception of children born SGA who had a mean IGF-I SDS of −0.7, mean values for IGF-I SDS were below −1.5 SDS for all indications at baseline. Mean GH doses by indication ranged from 0.036 (MPHD) to 0.049 (ISS) mg/kg/day (Table 1).

**Table 1:** Baseline characteristics and mean GH dose during two-year treatment period for total and pre-pubertal patient population by indication

	**Children with IGHD**		**Children born SGA**		**Children with ISS**		**Children with MPHD**	
	**Total**	**Pre-pubertal**	**Total**	**Pre-pubertal**	**Total**	**Pre-pubertal**	**Total**	**Pre-pubertal**
Total N (pre-pubertal % of total indication)	3,298	1,120 (34%)	678	434 (64%)	334	76 (23%)	272	165 (61%)
Male gender, N (%)	2,444 (74%)	778 (70%)	396 (58%)	274 (63%)	240 (72%)	59 (78%)	171 (63%)	106 (64%)
Mean chronological age ± SD (years)	10.2 ± 3.6	6.5 ± 2.8	7.9 ± 3.2	6.1 ± 2.1	10.9 ± 2.9	7.1 ± 2.3	7.9 ± 4.9	4.8 ± 3.5
Mean height SDS ± SD	−2.3 ± 1.0	−2.7 ± 1.0	−3.1 ± 0.9	−3.3 ± 0.9	−2.3 ± 0.8	−2.6 ± 0.9	−2.0 ± 1.5	−2.3 ± 1.6
Mean bone age delay from chronological age ± SD (years) [N]	−1.8 ± 1.4 [2,419]	−1.9 ± 1.3 [703]	−1.6 ± 1.4 [430]	−1.6 ± 1.2 [266]	−1.7 ± 1.5 [294]	−1.9 ± 1.4 [65]	−1.7 ± 1.8 [137]	−1.3 ± 1.7 [63]
Mean IGF-I SDS ± SD [N]	−1.8 ± 1.7 [2,784]	−1.4 ± 1.4 [890]	−0.7 ± 1.6 [434]	−0.5 ± 1.4 [276]	−1.6 ± 1.7 [308]	−1.2 ± 1.4 [66]	−1.9 ± 2.2 [222]	−1.4 ± 1.9 [133]
Mean GH dose during two-year treatment period ± SD (mg/kg/day) [N]	0.042 ± 0.013 [3,272]	0.038 ± 0.011 [1,110]	0.042 ± 0.013 [678]	0.041 ± 0.013 [434]	0.049 ± 0.012 [334]	0.046 ± 0.011 [76]	0.036 ± 0.013 [268]	0.035 ± 0.011 [163]

The percentage of pre-pubertal children in each indication group varied: ISS, 23%; IGHD, 34%; MPHD, 61%; and SGA, 64%. In comparison with the total population, the mean height SDS was lower for pre-pubertal children, although mean IGF-I SDS was higher in this group.

### Change in height

**Figure 1:**
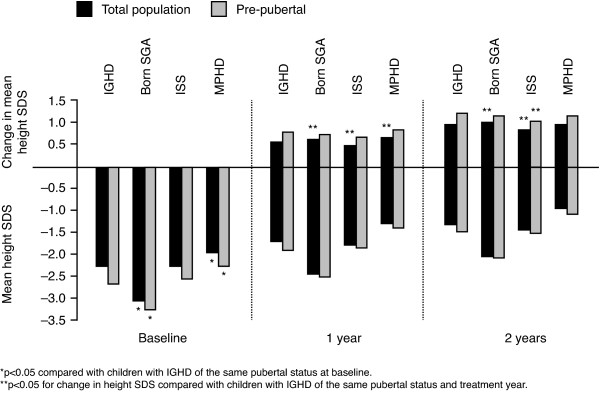
Mean height SDS at baseline, one year and two years and change in height SDS at one year and two years by indication.

In the total patient population, the mean change in height SDS at one year for all indications was +0.57 SDS or higher, except among children with ISS (+0.49 SDS; Figure 1). At one year, children with MPHD and children born SGA, responded with height SDS of +0.67 and +0.64, respectively; these height gains were +0.10 and +0.07 SDS greater than in children with IGHD (*p =* 0.001 for both). In contrast, height gain at one year with GH treatment in ISS was −0.08 SDS lower than that in IGHD (*p =* 0.005).

At two years of GH treatment, mean change in height from baseline was +0.99 vs. +0.97 SDS for patients with MPHD and IGHD, respectively, but was +1.03 SDS for patients born SGA (*p =* 0.047 vs. IGHD) and +0.84 for patients with ISS (*p <* 0.001 vs. IGHD). Normal height for age and gender (within ±2 SDS) was reached in 78% (IGHD), 45% (SGA), 76% (ISS), and 79% (MPHD) of children. Across all indications, 73% of patients reached normal height after two years of treatment.

In pre-pubertal children, the gains in height SDS at one year, and two years, were higher than in the total population (Figure 1). Among pre-pubertal children at one year, gain in height SDS did not differ significantly between IGHD and any other indication. At two years, the gain in height was above +1 SDS for all indications and comparable, except for in ISS compared with in IGHD (+1.04 vs. +1.24 SDS, *p =* 0.03) (Figure 1). Normal height was reached in 73% (IGHD), 46% (SGA), 72% (ISS), and 75% (MPHD) of children at two years in the pre-pubertal population.

### Change in IGF-I SDS

**Figure 2:**
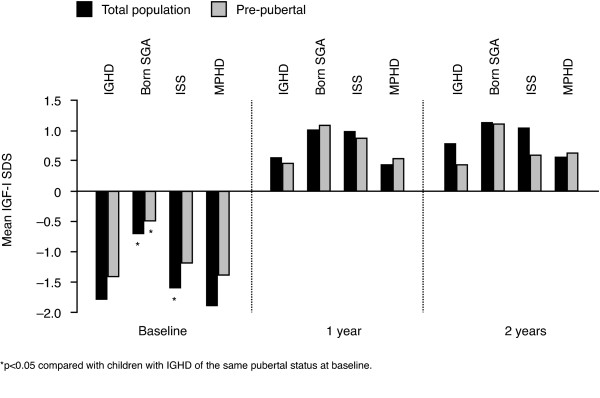
Mean IGF-I SDS at baseline, one year and two years by indication.

The mean change in IGF-I SDS for the total population was greater than +2 SDS after one and two years of GH treatment in all indications, except in children born SGA at one year (Figure 2). Mean values stayed within the reference range (±2 SDS) throughout the study. At two years, mean IGF-I SDS was 0.80 (IGHD), 1.15 (SGA), 1.06 (ISS), and 0.58 (MPHD). At one and two years of treatment, children born SGA had a significantly lower IGF-I increase than children with IGHD (+1.80 vs. +2.36 SDS, and +2.00 vs. +2.57 SDS, respectively, *p <* 0.001).

In the pre-pubertal population, the change in IGF-I only exceeded +2 SDS for ISS at 1 year (Figure 2). Between indications, no significant differences were observed in change in IGF-I SDS after either one or two years of GH treatment. Mean values for IGF-I SDS in pre-pubertal children were within the reference range for the two-year period, as in the total patient populations (Figure 2). At two years, mean IGF-I SDS was 0.45 (IGHD), 1.13 (SGA), 0.61 (ISS), and 0.65 (MPHD).

## Discussion

This analysis of international data from two large ongoing observational studies (NordiNet® IOS and NovoNet®/ANSWER Program®) is, to our knowledge, the first to compare growth and IGF-I response rates between children with IGHD and children with MPHD, SGA, and ISS treated with GH. In the total patient population, the two-year change in height SDS was approximately +1 SDS for each indication investigated. While the positive treatment responses for children with MPHD and children born SGA exceeded the change in height for children with IGHD at one year, only children born SGA had a higher response compared with IGHD after two years; these results are similar to those from other studies comparing outcomes with GH treatment in these indications [5,13,19]. It should be noted that although height change was expressed in SDS, which is generally considered more robust across gender and age than using change in cm, change in height SDS is not completely age-independent, because less variation is observed in height SD in younger vs. older ages [20]. This may, to a small degree, contribute to our finding that children with MPHD and those born SGA who were considerably younger at GH treatment start (mean 7.9 years) had a better growth response compared with IGHD (mean age of 10.2 years at treatment start). However, a positive effect of early age for growth hormone response is suggested as reported by others [5,13,19,21-25]. Among pre-pubertal children, where the differences in age were less, children with MPHD and children born SGA showed no significant difference in height gain compared with IGHD.

After two years of GH treatment, more than 75% of children with IGHD, MPHD, and ISS reached their normal height range (above −2 SDS). In contrast, although children born SGA experienced the highest growth response, only 45% reached normal height because their baseline height was considerably lower than any other group. The severe short stature of children born SGA observed in this study can be partially explained by the labeling in Europe, where patients must be below −2.5 SDS before treatment is initiated, and in France, where medical reimbursement is possible only if children born SGA are less than or equal to −3 SDS at treatment start.

Pre-pubertal children born SGA had the smallest increase in IGF-I SDS of all pre-pubertal indications, but had the highest baseline levels. A positive correlation has been proposed between increasing IGF-I levels and height increase in pre-pubertal children [26]. The results of our study appear to lend some support to this hypothesis, because pre-pubertal children in all other indications than ISS had comparable increases in IGF-SDS and height SDS.

The smallest two-year height SDS increase occurred in children with ISS (+0.84 SDS), which was significantly lower compared with IGHD. Several factors could have influenced the lower height increase seen in children with ISS: (a) age, the children with ISS in this study had a higher mean age at the start of GH treatment compared with the other indications investigated. Other studies have shown higher age at treatment initiation to be negatively associated with growth in children receiving GH, including ISS [16,21]; (b) pubertal status, the ISS group had the lowest percentage of pre-pubertal children (23%) (see later for discussion); (c) the variable etiology of the disease, because the diagnosis of ISS is based upon short stature due to a variety of unknown causes [27]. In our analysis, among the four indications, children with ISS had the highest mean age in the pre-pubertal subgroup (7.1 years at baseline) and a male gender dominance (78%), with bone age less or equally delayed in ISS compared with the three other indications studied (Table 1), which could suggest an underlying disorder of constitutional growth delay; d) lastly, in the total population, in spite of a higher GH dose given in ISS, the two-year IGF-I change was comparable between ISS and IGHD, while height gain was less in ISS. At baseline, IGF-I deficiency was less in ISS than in IGHD. These facts suggest some degree of GH or IGF-I insensitivity in ISS compared with IGHD, possibly influenced by differences in underlying disease nature, age, gender, and/or pubertal stage. Other studies support a degree of insensitivity to IGF-I in children with ISS [15,28], with the wide range of growth responses to GH in this patient population being consistent with the broad spectrum of genetic and molecular defects that result in IGF-I insensitivity [27].

Few other studies have compared the growth and/or IGF-I response to GH treatment between GHD and ISS. In a two-year, open-label trial, 63 children with GHD and 102 children with ISS were randomized to receive GH therapy based on an IGF-I target of 0 SDS, +2 SDS, or a dosing corresponding to the patient’s weight [15]. Children with GHD grew more than those with ISS in both IGF-targeted dosage groups despite having similar IGF-I levels. In the +2 SDS target group, the mean (±SD) change in height SDS for children with GHD was 2.04 (±0.17) compared with 1.33 (±0.09) for children with ISS. In the 0 SDS target group, the change in heights SDS results were 1.41 (±0.13) and 0.84 (±0.07), respectively. The results of a smaller clinical study comparing GHD with ISS were similar [16]. In spite of the real-life, heterogeneous nature of the observational studies reported here, our finding was consistent with a reduced growth response in ISS. However, all indications in this analysis surpassed the one-year response threshold for GH efficacy, which is generally considered to be approximately +0.25–0.5 SDS for change in height [2,22,29], thus demonstrating the ability of GH to stimulate linear growth regardless of indication.

For children with a variety of short stature indications, the height SDS at the onset of puberty correlates strongly with final adult height [9,10,13,21,23,24,27,30]. Correlations with adult height were not examined in this analysis, but pubertal status was found to have a marked influence on growth by both one and two years of treatment. In pre-pubertal children of all indication groups the change in height SDS was greater than in the total population, consistent with the preliminary two-year findings from the NovoNet®/ANSWER Program® [19], whose entirely US patient population overlapped with US patients included in this study when two-year growth data were available.

Further long-term follow-up with adult height data is needed to be able to describe fully the benefits of starting GH treatment early, especially in real-life situations, although long-term safety data on modern growth hormone therapy are generally reassuring [31]. To provide high-quality long term data, it is important to monitor the outcome of the populations in large observational studies, such as NordiNet® IOS and NovoNet®/ANSWER Program®, to assess the effectiveness and safety of GH therapy in children, especially in non-GHD indications.

Examining data from clinical practice contained in large observational studies like NordiNet® IOS and NovoNet®/ANSWER Program® can provide insights into optimal treatments in actual clinical practice, such as the desirability of starting growth hormone therapy before puberty observed in this study. It must, however, be considered that the differences in patient populations, diagnostic and treatment practices between countries represented in this large combined database may influence the outcome of any analysis. In particular, the results for IGF-I shown here should be treated with caution due to the lack of information on assay and control over local laboratory measurements and possible differences in normal IGF-I levels within the international patient cohort. Furthermore, although only a small variation was observed in mean GH dose in the total population across indications and also between the total population and pre-pubertal groups, it should be noted that dose recommendations for these indications vary from country to country and, indeed, even within-country variation in prescribing habits is likely. Lastly, selection bias from the entire cohort of children receiving GH treatment for any of the four indications studied could not be excluded; for example, drop outs could not be systematically analyzed.

## Conclusion

After two years of GH treatment, short children born SGA showed a greater height response than children with IGHD and MPHD (who experienced comparable growth responses), while children with ISS had a slightly lower response, possibly owing to confounders and/or differences in disease nature. Despite showing the greatest SDS height response, a lower number of SGA children reached a normal height range (above −2 SDS for mean) at the end of the two-year period due to their low baseline height. More than 75% of children with IGHD, ISS, and MPHD achieved a normal height range after two years of treatment. Beginning treatment at least two years before the onset of puberty was associated with an improved height gain, which suggests that GH treatment should start well in advance of puberty to optimize height growth outcomes.

## Competing interest

Viatcheslav Rakov is a former employee of Novo Nordisk Health Care AG.

Birgitte Tønnes Pedersen is an employee of Novo Nordisk A/S.

Peter A. Lee has participated in data collection for patient registries, with research support from Novo Nordisk, Pfizer, Ipsen, and Eli Lilly, and has served as a consultant for Novo Nordisk and Ipsen.

Judith Ross has participated in data collection for patient registries, with research support from Novo Nordisk, Pfizer, and Eli Lilly, and has served as a consultant for Novo Nordisk, Eli Lilly, and Abbot.

Peter A. Lee, Lars Sävendahl, Isabelle Oliver, Oliver Blankenstein, Judith Ross, Marta Snajderova and Henrik Thybo Christesen are members of the NordiNet® International Outcome Study International Study Committee.

Maithé Tauber is a Nordinet® International Outcome Study investigator.

Investigators received financial compensation from Novo Nordisk for time spent entering data on the electronic study forms.

## Authors’ contribution

PAL-Involved in assessment of database, deciding what factors would be analyzed, evaluating the assessment, and writing and revising the manuscript after review of previous related publications, LS- Involved in collecting data and assessment of database, deciding what factors would be analyzed, evaluating the assessment, and writing the manuscript, IO- Involved in collecting data and assessment of database, deciding what factors would be analyzed, evaluating the assessment, and writing the manuscript, MT- Involved in collecting data and assessment of database, deciding what factors would be analyzed, evaluating the assessment, and writing the manuscript, OB- Involved in collecting data and assessment of database, deciding what factors would be analyzed, evaluating the assessment, and writing the manuscript, JR- Involved in assessment of database, deciding what factors would be analyzed, evaluating the assessment, and writing the manuscript, MS- Involved in collecting data and assessment of database, deciding what factors would be analyzed, evaluating the assessment, and writing the manuscript, VR-Directed the project and oversaw the data analysis and intrepretation, BTP-Analyses of the data, including assessment of adequacy and statistics. HTC- Involved in collecting data and assessment of database, deciding what factors would be analyzed, evaluating the assessment, and writing the manuscript. All authors read and approved the final manuscript.

## Funding source

This study was funded by Novo Nordisk Health Care AG.
